# Integrative Network Pharmacology Prediction of the Mechanisms of Tri-Ka-Tuk: A Traditional Thai Herbal Formula

**DOI:** 10.2174/0118715303374703250429111617

**Published:** 2025-05-27

**Authors:** Pariyapat Singthong, Nopparat Songserm, Subramani Paranthaman Balasubramani, Watcharacha Krongkeha, Ananya Dechakhamphu

**Affiliations:** 1 Department of Health Sciences, Faculty of Public Health, Ubon Ratchathani Rajabhat University, Ubonratchathani 34000, Thailand;; 2 Thai Traditional Medicine Program, Faculty of Thai Traditional and Alternative Medicine, Ubon Ratchathani Rajabhat University, Ubonratchathani, 34000, Thailand;; 3 Department of Natural Sciences, Albany State University, Albany, GA, 31707, USA;; 4 Department of Food Processing and Culinary Science, Faculty of Science and Technology, Rajamangala University of Technology Rattanakosin, Nakhon Pathom, 73170, Thailand;; 5 Cosmetic Science and Spa Program, Faculty of Thai Traditional and Alternative Medicine, Ubon Ratchathani Rajabhat University, Ubonratchathani, 34000, Thailand

**Keywords:** Bioactive compounds, bioinformatics, herbal formula, traditional medicine, gene ontology, pathway enrichment analysis, protein-protein interaction (PPI) network

## Abstract

**Introduction:**

Tri-Ka-Tuk, a traditional Thai herbal formula composed of ginger, black pepper, and long pepper, has long been utilized for its therapeutic properties, particularly in promoting digestive health, enhancing metabolism, and reducing inflammation. However, the underlying molecular mechanisms remain poorly understood. This study employs an integrative network pharmacology approach to reveal the bioactive compounds and pathways mediating the formula’s therapeutic effects.

**Methods:**

Bioactive compounds of Tri-Ka-Tuk were identified through literature review and database searches. Targets were predicted using SwissTargetPrediction, and gene ontology (GO) enrichment analysis was conducted *via* ShinyGO. Pathway enrichment analysis utilized KEGG databases to map associated signaling pathways. Protein-protein interaction (PPI) networks were constructed using the STITCH database.

**Results:**

GO analysis revealed that Tri-Ka-Tuk's targets are enriched in biological processes such as protein autophosphorylation and phosphorylation-dependent signaling. Cellular component enrichment indicated involvement in membrane-associated regions critical for signaling. Molecular function analysis highlighted kinase activity and ATP binding as central mechanisms. Pathway enrichment analysis identified significant pathways, including the AGE-RAGE signaling pathway in diabetic complications, the HIF-1 pathway, and cancer-related pathways. PPI network analysis showed that hub proteins such as VEGFA, CDK1, and MAPK1 play key roles in mediating the formula’s effects.

**Conclusion:**

The findings suggest that Tri-Ka-Tuk exerts its therapeutic effects by modulating key molecular pathways involved in inflammation, oxidative stress, apoptosis, and metabolism. The integrative network pharmacology approach followed in this research bridges traditional knowledge with modern pharmacological insights, and highlights Tri-Ka-Tuk's potential as a therapeutic agent for managing diabetes, cancer, and metabolic disorders. Experimental validation of these predictions is essential to confirm the efficacy of the formulation and to expand its clinical applications.

## 1 INTRODUCTION

Tri-Ka-Tuk is a traditional Thai herbal formula consisting of three key ingredients: ginger (*Zingiber officinale*), black pepper (*Piper nigrum*), and long pepper (*Piper retrofractum*). This combination has been widely used in traditional Thai medicine (TTM) due to its potent effects on digestion, metabolism, and overall wellness. Historically, Tri-Ka-Tuk has been employed in Thai medicine to treat a range of ailments, primarily focusing on improving digestive health and enhancing metabolic functions [1]. The combination of these three pungent herbs is used to balance the body's elements, particularly the fire element, which governs digestion and metabolism in Thai medical theory. Tri-Ka-Tuk is also considered effective in alleviating respiratory symptoms such as coughs, colds, and congestion due to its warming and stimulating properties [2]. The formula is often prescribed for individuals experiencing sluggish digestion, bloating, or symptoms associated with the common cold. Its role in enhancing circulation and metabolism has made it a cornerstone of traditional remedies aimed at boosting vitality and energy. This blend reflects TTM’s holistic approach, where herbs are used not just for symptom relief but for maintaining balance and preventing disease [2]. Recent studies have explored the therapeutic applications of Tri-Ka- Tuk, a formulation consisting of *P. nigrum*, *P. retrofractum*, and *Z. officinale*, in various disease models. HPLC analysis has identified piperine as a major active constituent, while antioxidant assays and cytotoxic studies suggest potential anti-cancer activity, particularly against cholangiocarcinoma cells, *via* cell cycle arrest and modulation of CDK2, p53, p21, and p27 expression [3]. Additionally, Tri-Ka-Tuk has demonstrated antioxidant properties, alpha-glucosidase inhibitory effects, and metabolic regulation, indicating potential use in diabetes management [4]. Moreover, essential oil formulations of Tri-Ka-Tuk have been explored to enhance bioavailability and therapeutic effectiveness, particularly in aromatherapy and wellness applications [5]. These findings collectively support the traditional uses of Tri-Ka-Tuk while providing scientific evidence for its integration into modern pharmacology.

The precise molecular mechanisms behind the effectiveness of traditional herbal formulas like Tri-Ka-Tuk remain largely underexplored. Herbal formulas like Tri-Ka-Tuk consist of multiple bioactive compounds, each interacting with numerous biological targets. The synergistic or antagonistic effects of these compounds are difficult to pinpoint, making it challenging to map out their precise molecular actions within the body. While traditional use provides evidence of effectiveness, rigorous molecular studies and clinical trials for many herbal formulas are lacking. Without well-designed studies, it is difficult to identify the molecular targets of individual compounds and how they influence biological pathways. Many mechanisms remain speculative based on the known pharmacology of individual components rather than comprehensive analyses of the complete formula.

Recent advances in network pharmacology and systems biology provide powerful tools to study these interactions by integrating multiple datasets, such as gene expression profiles, protein-protein interactions, and metabolomic analyses. Unlike conventional pharmacological models that focus on single targets, network pharmacology enables the identification of multi-target interactions and key regulatory hubs within complex biological networks, offering a more holistic understanding of the mechanisms of herbal medicine [6-11].

Studies from 2022–2024 have demonstrated how network pharmacology can predict active compounds, identify core molecular pathways, and validate synergistic effects within multi-component herbal formulations, including those used in traditional Thai and Chinese medicine [12-23]. Furthermore, multi-omics approaches, including transcriptomics, proteomics, and metabolomics, are being increasingly used to reconcile traditional knowledge with modern pharmacology [24-26]. These approaches allow for the systematic identification of pharmacological mechanisms, biomarkers of efficacy, and potential safety concerns [27].

By mapping the interactions between compounds and biological networks, network pharmacology offers a holistic view of how these interactions influence disease processes. This approach enables the identification of key nodes or targets within a network that may contribute to the therapeutic effects of herbal formulas [14, 28-58]. Importantly, it provides a means to study the synergistic effects of multiple compounds working together, which is critical for understanding the mechanisms of multi-component traditional herbal medicines like Tri-Ka-Tuk. With the advent of large-scale data analysis tools, including genomics, proteomics, and metabolomics, network pharmacology leverages big data to predict molecular interactions, therapeutic targets, and disease pathways. This approach has the potential to transform traditional herbal medicine research by providing scientific validation for herbal formulations and facilitating the discovery of novel therapeutic strategies [59, 60].

By applying integrative network pharmacology, researchers can systematically identify and predict the interactions between the bioactive compounds in Tri-Ka-Tuk and various biological pathways implicated in health and disease. This approach not only uncovers the individual contributions of each herb but also reveals potential synergistic effects that enhance the formula's overall efficacy. Furthermore, network pharmacology can pinpoint key molecular targets, such as receptors, enzymes, or signaling pathways, involved in digestive and metabolic processes, inflammation, and immune regulation. Through this advanced methodology, the study aims to bridge the gap between traditional medicine and modern pharmacological insights. By elucidating the bioactive compounds and their mechanisms of action, network pharmacology can validate the therapeutic benefits of Tri-Ka-Tuk and potentially lead to the discovery of novel drug candidates or therapeutic strategies for digestive and metabolic disorders.

The objective of this study is to apply integrative network pharmacology to predict the molecular mechanisms underlying the therapeutic effects of the Tri-Ka-Tuk formula. By systematically identifying the interactions between the bioactive compounds in Tri-Ka-Tuk and their corresponding pharmacological targets and pathways, this study aims to uncover the synergistic actions responsible for its therapeutic efficacy. Ultimately, this research seeks to unite traditional knowledge with modern pharmacological insights, providing a scientific foundation for the therapeutic application and potential drug development of the Tri-Ka-Tuk formula. The results of the study will help to further develop the Tri-Ka-Tuk formula and its potential use as an alternative medicine in the future.

## 2 MATERIALS AND METHODS

### 2.1 Data Collection and Preparation

The bioactive compounds in the Tri-Ka-Tuk formula (*Z. officinale*, *P. nigrum*, and *P. retrofractum*) were identified through literature review and database searches, focusing on their known pharmacological properties. These compounds were further analyzed to predict their interactions with molecular targets and biological pathways. The list of bioactive compounds used for analysis is detailed in Table **1** [61-71].

### 2.2 Target Identification

The target identification was conducted by obtaining the canonical SMILES of bioactive compounds and submitting them to the SwissTargetPrediction online tool (accessed on 15^th^ November 2024) [71]. Targets were selected based on a probability score greater than 0.1. SwissTargetPrediction assigns probability scores based on chemical similarity to known ligands, and a cutoff of 0.1 ensures that only meaningful interactions with a high likelihood of occurrence are retained while minimizing false positives. Targets with very low scores are likely to be random associations rather than biologically relevant interactions. This threshold has been used in previous network pharmacology studies to maintain a reliable prediction of potential protein-ligand interactions [72-77].

### 2.3 Gene Ontology and Pathway Enrichment Analysis

Gene Ontology (GO) and pathway enrichment investigations were performed *via* ShinyGO (version 0.81), an online platform for enrichment analysis across various species [78-80]. The analysis aimed to identify biological processes, molecular functions, and cellular components related to the targets of chemicals in Tri-Ka-Tuk. GO terms and KEGG pathways with a false discovery rate (FDR) below 0.05 were deemed significantly enriched.

### 2.4 Protein-Protein Interaction (PPI) Network Construction

The STITCH database (version 5.0) was used for compound-protein interaction network analysis, with a confidence score threshold of >0.7 to ensure high-confidence interactions [81].

## 3 RESULTS

### 3.1 Enrichment of Cellular Biological Processes with the Predicted Targets of Tri-Ka-Tuk

**Fig. (1):**
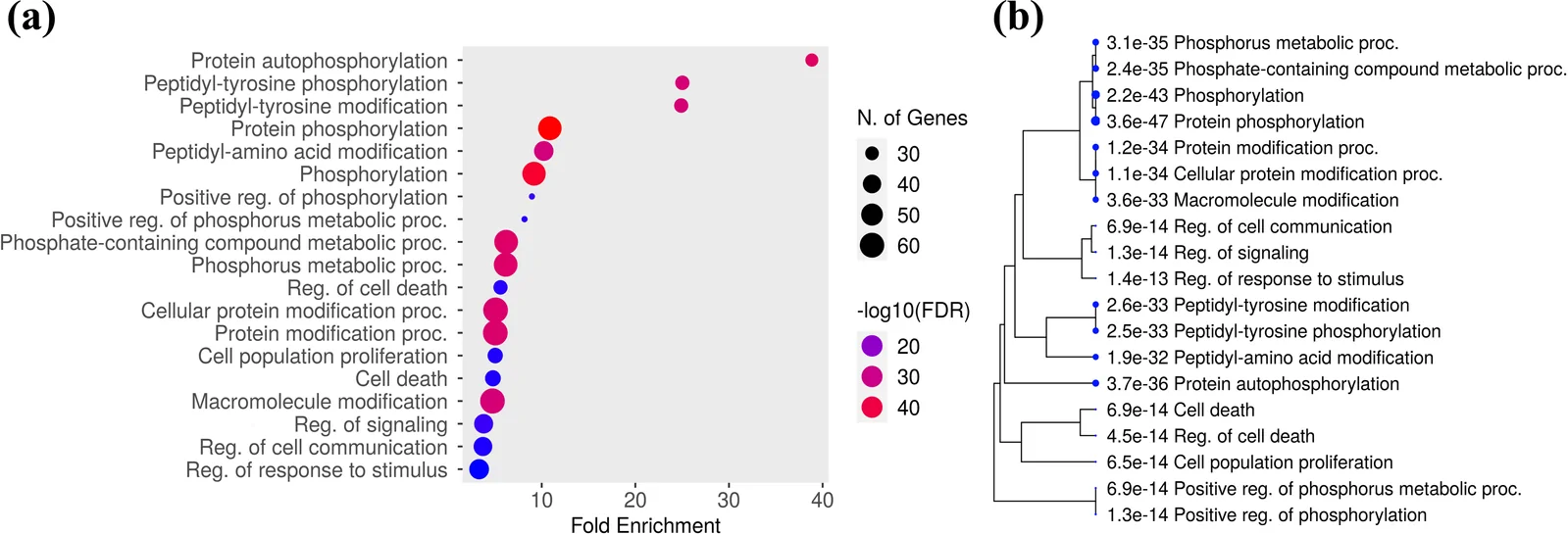
The Gene Ontology (GO) enrichment analysis for biological processes associated with the Tri-Ka-Tuk herbal formula's predicted targets. (**a**) Bubble plot showing fold enrichment, gene count (bubble size), and -log10(FDR) (color). (**b**) Hierarchical clustering of enriched GO terms with corresponding FDR values.

The GO enrichment analysis revealed significant biological processes associated with the predicted targets of the Tri-Ka-Tuk herbal formula (Fig. **1**). The bubble plot (Fig. **1a**) highlights key biological processes, with fold enrichment values indicating the degree of overrepresentation. The bubble size represents the number of genes involved in each process, while the color gradient denotes the significance level as −log_10_(FDR). Among the most enriched processes were “protein autophosphorylation,” “peptidyl-tyrosine phosphorylation,” “protein phosphorylation,” and “phosphorus metabolic processes,” all of which are integral to cellular signaling and metabolic regulation.

Hierarchical clustering of GO terms (Fig. **1b**) demonstrated functional relationships between enriched processes, forming clusters such as “cellular protein modification processes” and “positive regulation of phosphorus metabolic processes.” These clusters highlight the interconnected roles of these processes in protein modification, signal transduction, and cellular regulation. The associated FDR values further validate the statistical significance of these pathways.

Overall, these findings suggest that the Tri-Ka-Tuk formula exerts its effects by modulating pathways involved in phosphorylation, metabolic regulation, and cell signaling, supporting its potential role in therapeutic applications targeting these mechanisms.

### 3.2 Enrichment of Cellular Components Associated with the Predicted Targets of Tri-Ka-Tuk

**Fig. (2):**
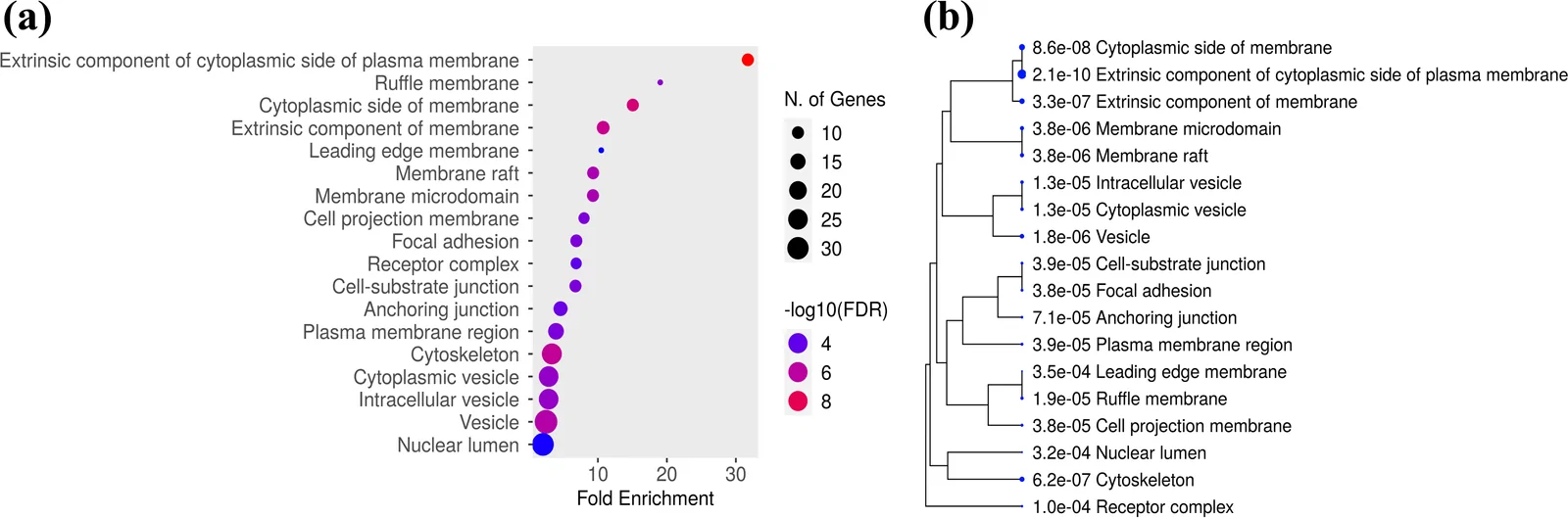
Gene Ontology (GO) enrichment analysis for cellular components associated with the predicted targets of the Tri-Ka-Tuk herbal formula. (**a**) Bubble plot showing fold enrichment, gene count (bubble size), and -log10(FDR) (color). (**b**) Hierarchical clustering of enriched GO terms with FDR values.

The GO enrichment analysis for cellular components identified significant localization patterns for the predicted targets of the Tri-Ka-Tuk herbal formula (Fig. **2**). Key enriched compartments include the “extrinsic component of the cytoplasmic side of the plasma membrane,” “ruffle membrane,” “cytoplasmic side of the membrane,” and “membrane microdomain,” indicating a strong association with cellular signaling and structural dynamics (Fig. **2a**). The hierarchical clustering of GO cellular components (Fig. **2b**) further illustrates the relationships among enriched compartments. Notable clusters include “cytoplasmic vesicle” and “focal adhesion,” as well as other critical regions such as the “cell-substrate junction” and “anchoring junction,” which are essential for cellular communication and structural integrity. The FDR values highlight the statistical significance of these findings, emphasizing their relevance in the mechanistic roles of the formula's bioactive compounds. These results suggest that the Tri-Ka-Tuk formula influences key cellular compartments involved in signaling, adhesion, and vesicular transport, further supporting its potential therapeutic applications through modulation of these cellular structures and assemblies.

### 3.3 Enrichment of Molecular Functions Associated with the Predicted Targets of Tri-Ka-Tuk

**Fig. (3):**
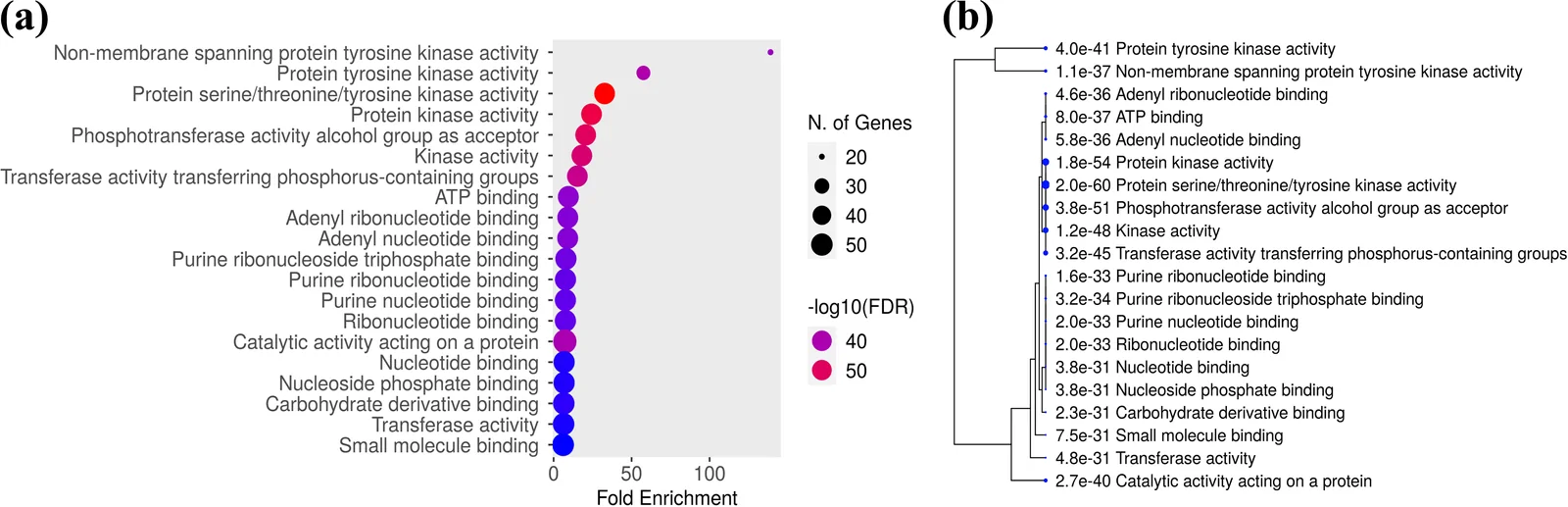
Gene Ontology (GO) enrichment analysis for molecular functions associated with the predicted targets of the Tri-Ka-Tuk herbal formula. (**a**) Bubble plot showing fold enrichment, gene count (bubble size), and -log10(FDR) (color). (**b**) Hierarchical clustering of enriched GO terms with FDR values.

The GO enrichment analysis for molecular functions highlights significant biochemical activities associated with the predicted targets of the Tri-Ka-Tuk herbal formula (Fig. **3**). The most enriched functions include “protein tyrosine kinase activity,” “protein serine/threonine/tyrosine kinase activity,” “ATP binding,” and “transferase activity transferring phosphorus-containing groups,” underscoring their central roles in enzymatic and signaling processes (Fig. **3a**). The hierarchical clustering of GO molecular functions (Fig. **3b**) demonstrates the relationships among enriched functions, grouping related activities such as “kinase activity,” “nucleotide binding,” and “adenyl ribonucleotide binding.” These clusters indicate the functional interconnectivity of the predicted targets in phosphorylation-dependent signaling pathways and energy metabolism. The FDR values further substantiate the statistical relevance of these molecular functions. These results suggest that the Tri-Ka-Tuk formula targets molecular functions essential for phosphorylation, nucleotide metabolism, and protein modification, supporting its potential role in regulating cellular signaling and enzymatic activity.

### 3.4 Pathway Enrichment Analysis of Tri-Ka-Tuk Targets

**Fig. (4):**
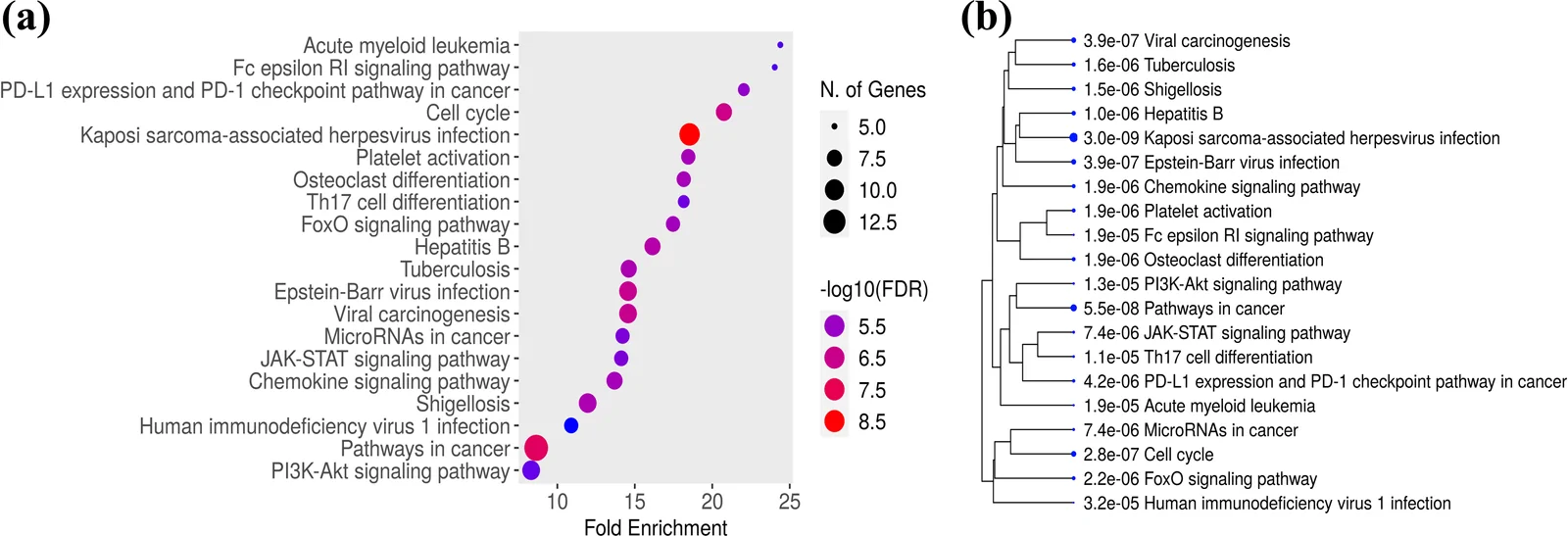
KEGG pathway enrichment analysis for the predicted targets of the Tri-Ka-Tuk herbal formula. (**a**) Bubble plot showing fold enrichment, gene count (bubble size), and -log10(FDR) (color). (**b**) Hierarchical clustering of enriched GO terms with FDR values.

The KEGG pathway enrichment analysis identified significant signaling pathways and biological processes associated with the predicted targets of the Tri-Ka-Tuk herbal formula (Fig. **4**). Prominent enriched pathways include “PD-L1 expression and PD-1 checkpoint pathway in cancer,” “Fc epsilon RI signaling pathway,” “acute myeloid leukemia,” and “Kaposi sarcoma-associated herpesvirus infection,” highlighting their relevance in immune modulation, cancer biology, and infectious diseases (Fig. **4a**). The hierarchical clustering of pathways (Fig. **4b**) reveals functional relationships among the enriched pathways. Clusters such as “PI3K-Akt signaling pathway,” “JAK-STAT signaling pathway,” and “chemokine signaling pathway” emphasize the involvement of immune and inflammatory responses, while pathways like “cell cycle” and “FoxO signaling” underscore roles in cellular proliferation and survival. The associated FDR values underscore the statistical significance of these pathways. These findings suggest that the Tri-Ka-Tuk formula exerts its effects through the modulation of critical signaling pathways involved in immune regulation, cancer progression, and pathogen responses, supporting its potential therapeutic applications in these domains.

**Fig. (5):**
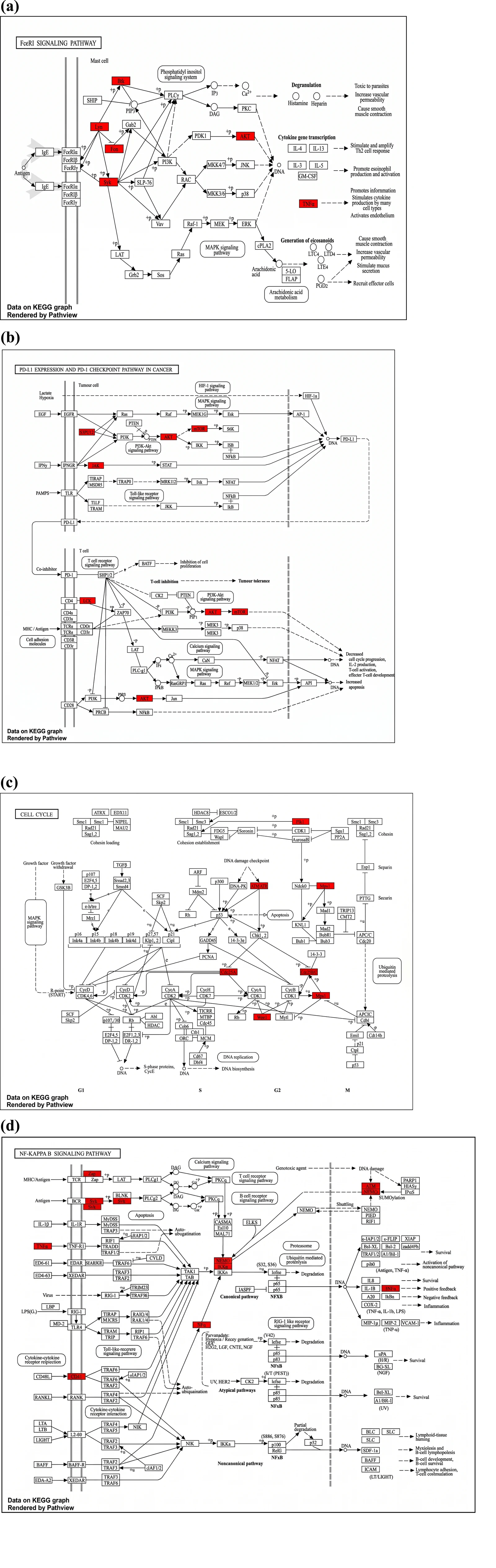
Pathway mapping of the predicted targets of the Tri-Ka-Tuk herbal formula. (**a**) FcRI signaling pathway. (**b**) PD-L1 expression and PD-1 checkpoint pathway in cancer. (**c**) Cell cycle. (**d**) NF-kappa B signaling pathway. Red boxes indicate mapped target proteins.

Pathway mapping of the predicted targets of the Tri-Ka-Tuk herbal formula revealed significant involvement in multiple signaling pathways (Fig. **5**). Highlighted targets are marked in red to indicate their association with key nodes within each pathway. In Fig. (**5a**), the Fc epsilon RI signaling pathway demonstrates the formula's potential to regulate immune responses by activating downstream signaling cascades, including MAPK signaling and degranulation processes, which are critical for mast cell-mediated immune defense. In Fig. (**5b**), the PD-L1 expression and PD-1 checkpoint pathway in cancer illustrates the formula's role in modulating immune checkpoint mechanisms, particularly through targets associated with T-cell activation and tumor immune tolerance, suggesting its potential impact on tumor immunity. In Fig. (**5c**), the cell cycle pathway highlights the involvement of the formula in regulating critical checkpoints, such as DNA replication and cell division, emphasizing its potential effects on cellular proliferation and genomic stability. In Fig. (**5d**), the NF-κB signaling pathway showcases the regulation of pro-inflammatory and survival signals, with predicted targets associated with cytokine production, apoptosis, and immune response modulation, underscoring its relevance in inflammatory and immune-related diseases.

These findings support the hypothesis that the Tri-Ka- Tuk formula exerts its therapeutic effects by targeting pathways involved in immune regulation, cancer progression, cell cycle control, and inflammatory signaling, demonstrating its broad pharmacological potential.

### 3.5 The Protein-Protein Interaction (PPI) Network Analysis

**Fig. (6):**
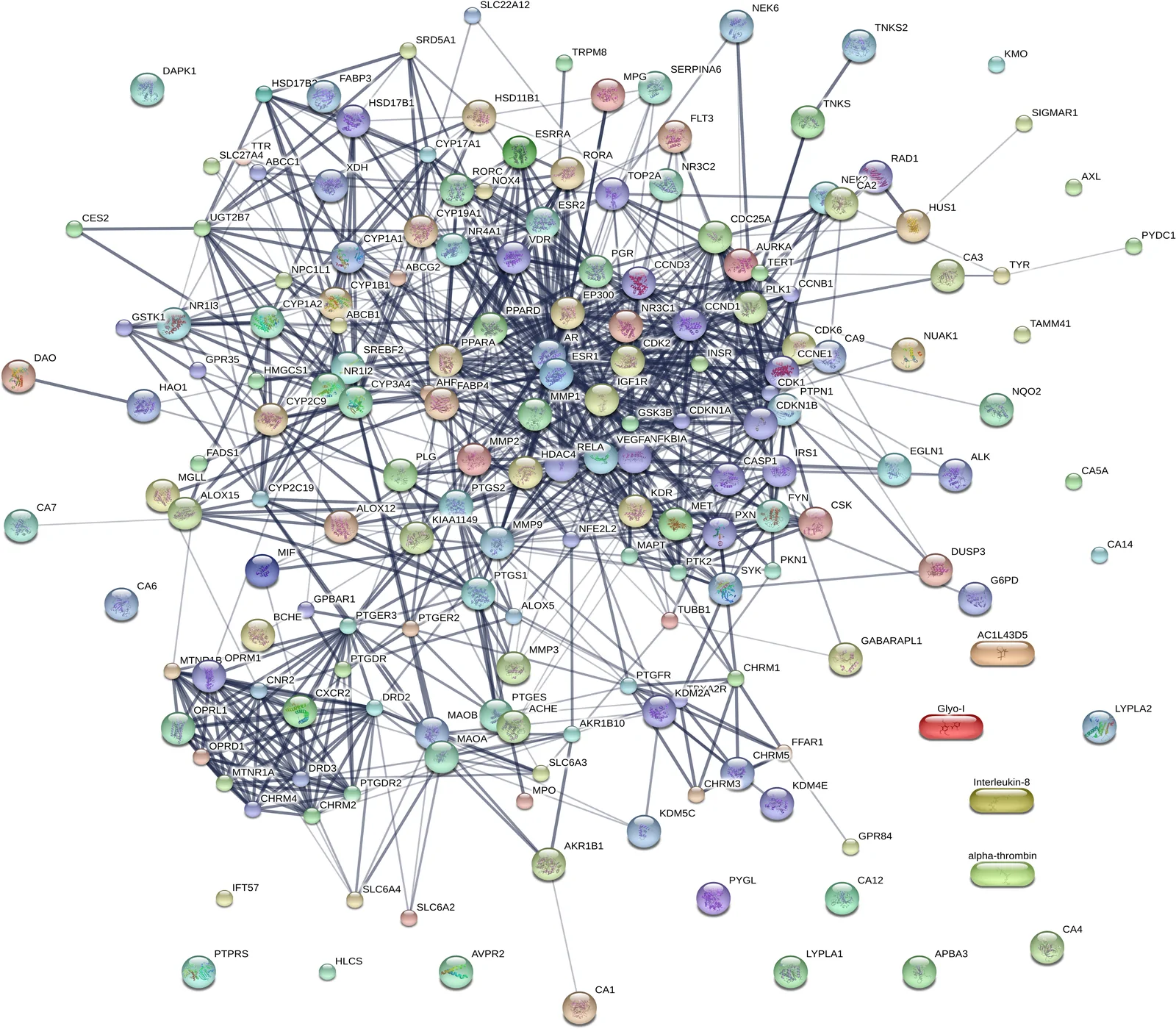
Protein-protein interaction (PPI) network analysis of the predicted targets of the Tri-Ka-Tuk herbal formula.

The protein-protein interaction (PPI) network of the predicted targets for the Tri-Ka-Tuk herbal formula reveals a highly interconnected network, indicating extensive interactions among the involved proteins (Fig. **6**). Each node in the network represents a protein, while the edges denote interactions based on functional or physical associations. Proteins with higher connectivity, or hub proteins, are prominently centralized within the network, suggesting their critical roles in mediating the biological effects of the formula. Several key hubs, such as CDK1, VEGFA, PPARA, and MAPK1, are observed, highlighting their involvement in essential pathways, including cell cycle regulation, angiogenesis, lipid metabolism, and signal transduction. The clustering of nodes into functional modules indicates groups of proteins working collaboratively within specific pathways or biological processes. Additionally, the peripheral nodes represent less-connected proteins, which may play specialized roles or interact with hub proteins to facilitate broader network functions. The dense connectivity observed within the network underscores the potential multi-target and multi-pathway mechanisms of the Tri-Ka-Tuk formula, emphasizing its pharmacological complexity. These findings demonstrate that the formula likely exerts its therapeutic effects through the modulation of a network of proteins, targeting multiple pathways to achieve a synergistic impact on biological systems.

Proteins with high closeness centrality are key regulators in biological pathways, as they facilitate efficient signal transduction within the network. Our analysis identifies CDK1, VEGFA, and MAPK1 as hub proteins with high closeness scores, indicating their potential roles in mediating Tri-Ka-Tuk’s therapeutic effects. CDK1 is a critical regulator of the cell cycle, suggesting that Tri-Ka-Tuk may influence cell proliferation and apoptosis. VEGFA, a major factor in angiogenesis, indicates a possible role in vascular health and inflammation regulation. MAPK1 is a central kinase involved in multiple signaling pathways, including those related to immune response, stress adaptation, and cellular growth. Their high closeness centrality suggests that they serve as central connectors, integrating various biochemical signals and amplifying the biological effects of the herbal formulation. These findings strengthen the hypothesis that Tri-Ka-Tuk exerts its pharmacological actions through a multi-target network mechanism, reinforcing the importance of experimental validation to confirm these interactions. Further studies should investigate how the modulation of these hub proteins translates into therapeutic benefits, particularly in disease models related to inflammation, metabolic disorders, and immune regulation.

## 4 DISCUSSION

The integrative network pharmacology analysis of the Tri-Ka-Tuk herbal formula provides comprehensive insights into its multi-target and multi-pathway mechanisms of action. The enrichment of biological processes, cellular components, molecular functions, signaling pathways, and protein-protein interactions underscores the formula's pharmacological complexity and therapeutic potential.

The enrichment of biological processes reveals the formula's strong association with pathways involved in phosphorylation, metabolic regulation, and cell signaling. Key processes, such as “protein autophosphorylation,” “peptidyl- tyrosine phosphorylation,” and “protein phosphorylation,” highlight its potential to modulate critical signaling cascades that underpin cellular communication and metabolic homeostasis. These findings align with the traditional use of herbal medicines to regulate cellular function and address metabolic disorders [82-84].

In terms of cellular localization, the analysis identified significant involvement of cellular components associated with signaling and structural integrity, such as the “extrinsic component of the cytoplasmic side of the plasma membrane” and “cytoplasmic vesicle.” These enrichments suggest that the formula influences key compartments essential for cellular signaling, adhesion, and vesicular transport. The involvement of focal adhesion and membrane microdomains further supports the hypothesis that the formula modulates cellular communication and structural organization [85, 86].

The molecular function analysis highlights the formula's capacity to regulate critical biochemical activities, including kinase activity, ATP binding, and transferase functions. These findings point to the formula's potential to regulate phosphorylation-dependent pathways, energy metabolism, and enzymatic activities, which are essential for cellular survival, proliferation, and homeostasis [87, 88].

Pathway enrichment and mapping analysis identified multiple critical signaling pathways influenced by the formula, including the “PD-L1 expression and PD-1 checkpoint pathway,” “Fc epsilon RI signaling pathway,” “cell cycle,” and “NF-κB signaling pathway.” These pathways play pivotal roles in immune regulation, inflammation, cancer progression, and cellular proliferation. The clustering of pathways involved in immune responses, such as the “PI3K-Akt signaling pathway” and “JAK-STAT signaling pathway,” underscores the formula's potential to modulate immune and inflammatory mechanisms. The enrichment of pathways related to cancer biology and pathogen responses supports its application in oncology and the prevention and control of infectious diseases.

The protein-protein interaction (PPI) network analysis further reinforces the multi-target nature of the Tri-Ka-Tuk formula. Central hub proteins such as CDK1, VEGFA, PPARA, and MAPK1 demonstrate high connectivity and functional importance within the network, suggesting their roles as key mediators of the formula's therapeutic effects. The dense connectivity and clustering of proteins into functional modules highlight the synergistic interactions within the network, allowing the formula to exert broad and complementary effects on multiple biological systems.

The pharmacokinetic characteristics of herbal bioactive substances are essential for evaluating their medicinal effectiveness. In the case of Tri-Ka-Tuk, three principal bioactive compounds—piperine, gingerol, and piperlongumine—have been thoroughly investigated regarding their absorption, distribution, metabolism, and excretion (ADME) properties. Piperine, a major alkaloid from black pepper, has been shown to have moderate oral bioavailability due to its poor water solubility and first-pass metabolism. However, it significantly enhances the bioavailability of other co-administered compounds by inhibiting cytochrome P450 (CYP3A4) enzymes and P-glycoprotein (P-gp) efflux transporters [89-93]. This property makes piperine an important bioenhancer in herbal formulations, increasing the systemic absorption of polyphenols and alkaloids. Gingerol, the principal bioactive compound in ginger, exhibits moderate metabolic stability but undergoes extensive phase I and phase II metabolism in the liver, leading to the formation of shogaol and glucuronidated metabolites. Studies indicate that gingerol is rapidly absorbed but has a short half-life due to rapid biotransformation. However, its metabolites retain significant biological activity, contributing to its pharmacological effects in inflammation and metabolic regulation [94-96]. Piperlongumine, derived from long pepper, has gained attention for its selective cytotoxicity in cancer cells and anti-inflammatory effects [97-101]. It exhibits high lipophilicity, which facilitates cellular uptake, but it is also subject to metabolic modifications *via* glutathione conjugation and cytochrome P450 enzymes [102-104]. Due to its structural properties, piperlongumine demonstrates reasonable plasma stability, but further studies on its metabolism and excretion pathways are necessary to optimize its therapeutic potential [105, 106]. These ADME characteristics suggest that the combined presence of these bioactive compounds in Tri-Ka-Tuk may contribute to a synergistic effect, enhancing therapeutic efficacy through improved absorption and metabolic interactions. Future pharmacokinetic studies should focus on quantifying systemic exposure, metabolic fate, and tissue distribution of these bioactive compounds in both preclinical and clinical models to ensure optimal therapeutic outcomes.

Tri-Ka-Tuk’s bioactive compounds, particularly piperine, gingerol, and piperlongumine, exhibit strong anti-inflammatory properties by modulating pathways such as the nuclear factor-kappa B (NF-κB) signaling pathway. NF-κB plays a central role in inflammation by regulating the expression of cytokines, chemokines, and adhesion molecules involved in immune responses. By downregulating NF-κB activation, Tri-Ka-Tuk may reduce chronic inflammation, which is a common underlying factor in metabolic disorders and cancer progression [107-109].

Moreover, inflammation is closely linked to metabolic dysregulation, particularly through the AGE-RAGE signaling pathway and AMP-activated protein kinase (AMPK) signaling. The AGE-RAGE pathway is implicated in oxidative stress and insulin resistance, conditions that contribute to metabolic disorders such as diabetes and obesity [110-112]. By inhibiting AGE-RAGE signaling, Tri-Ka-Tuk’s bioactive compounds may help mitigate oxidative stress and improve insulin sensitivity. Simultaneously, AMPK activation plays a crucial role in cellular energy homeostasis, promoting glucose uptake and lipid metabolism. Tri-Ka-Tuk may enhance metabolic regulation through AMPK activation, which in turn reduces inflammation and improves mitochondrial function.

Apoptosis, or programmed cell death, is another key mechanism influenced by Tri-Ka-Tuk, particularly in the context of cancer therapy. The formula’s bioactive components have been shown to regulate apoptotic pathways, such as the mitogen-activated protein kinase (MAPK) pathway and p53-mediated apoptosis [113-116]. MAPK signaling integrates inflammatory responses with cell proliferation and apoptosis, indicating that Tri-Ka-Tuk may exert a dual role in suppressing tumor growth while reducing inflammation. Additionally, p53, a well-known tumor suppressor, can be upregulated by Tri-Ka-Tuk’s active constituents, leading to cell cycle arrest and apoptosis in cancer cells.

The interplay between these pathways suggests that Tri-Ka-Tuk exerts its pharmacological effects through a multi-targeted approach, simultaneously modulating inflammation, metabolism, and apoptosis to restore homeostasis. The ability of Tri-Ka-Tuk to target these interconnected pathways highlights its potential as a holistic therapeutic agent, particularly for conditions such as metabolic syndrome, cancer, and chronic inflammatory diseases. Future studies should be conducted to validate these mechanistic interactions through* in vitro *and *in vivo* studies and multi-omics approaches to delineate the precise molecular mechanisms underlying Tri-Ka-Tuk’s therapeutic effects.

Although network pharmacology provides valuable insights into the potential mechanisms of action of herbal formulations like Tri-Ka-Tuk, it has inherent limitations that must be acknowledged. Network-based predictions rely on databases, computational models, and algorithms that integrate existing biological and pharmacological knowledge. However, these models often lack comprehensive experimental validation, leading to potential biases and limitations in accurately predicting real-world interactions. One major limitation is that network pharmacology typically considers known drug-target interactions and curated pathway data, which may not account for novel bioactive compounds or unknown mechanisms of action. Additionally, computational predictions do not consider factors such as compound bioavailability, metabolism, or potential off-target effects, which are crucial for therapeutic efficacy. To further strengthen the translational impact of our findings, an integrated multi- omics approach is essential. Genomics, transcriptomics, proteomics, and metabolomics can provide deeper insights into how Tri-Ka-Tuk influences multiple biological pathways and networks. By analyzing changes in gene expression, protein interactions, and metabolite alterations, we can obtain a system-wide understanding of the herbal formula’s therapeutic mechanisms. Furthermore, molecular docking simulations offer a predictive framework for understanding ligand-protein interactions at the atomic level. Docking studies on piperine, gingerol, and piperlongumine suggest strong binding affinities with CDK1, VEGFA, and MAPK1, supporting their involvement in key disease-modulating pathways. However, molecular dynamics (MD) simulations are needed to evaluate the stability of these interactions in dynamic physiological environments. Targeted experimental validation remains a critical next step.* In vitro *studies using relevant cell lines will confirm the biological activity of the predicted interactions, while pharmacokinetic studies will assess the absorption, distribution, metabolism, and excretion (ADME) of these bioactive compounds. Further validation in animal models will provide insights into therapeutic efficacy, safety, and potential toxicity. Ultimately, clinical studies are required to translate these findings into real- world applications. Carefully designed randomized controlled trials (RCTs) will help determine the efficacy and safety of Tri-Ka-Tuk in human populations. Combining multi-omics, computational modeling, and experimental validation will ensure a comprehensive and scientifically rigorous evaluation of this traditional Thai herbal formula, paving the way for future integration into modern medicine.

Chromatography-mass spectrometry (MS) plays a crucial role in accurately identifying and quantifying the bioactive compounds present in herbal formulations such as Tri-Ka-Tuk. While HPLC (high-performance liquid chromatography) and GC-MS (gas chromatography-mass spectrometry) have been used in some studies to detect piperine and gingerol, a more comprehensive metabolic profiling approach is required to fully characterize the diverse phytochemical constituents of this herbal blend. Advanced techniques such as LC-MS/MS (liquid chromatography-tandem mass spectrometry) and UPLC-QTOF-MS (ultra-performance liquid chromatography quadrupole time-of-flight mass spectrometry) provide superior sensitivity, resolution, and structural elucidation capabilities, enabling the detection of minor bioactive components that may contribute to Tri-Ka-Tuk’s therapeutic efficacy. Furthermore, chromatography-mass spectrometry is essential for quality control, batch standardization, and pharmacokinetic studies. Variations in bioactive compound concentrations due to plant source, harvesting conditions, or extraction methods can influence the pharmacological activity of Tri-Ka-Tuk. By implementing high-throughput metabolomic profiling, future studies can ensure batch-to-batch consistency and optimize extraction techniques to maximize the bioavailability of key phytochemicals. Additionally, metabolomic fingerprinting using MS-based methods will allow for a deeper investigation into the interactions between Tri-Ka-Tuk compounds and endogenous metabolites, providing insights into potential synergistic or antagonistic effects at the metabolic level. This comprehensive analysis will support the scientific validation and regulatory approval of Tri-Ka-Tuk as a standardized herbal formulation.

Overall, these findings emphasize the pharmacological complexity of the Tri-Ka-Tuk herbal formula, demonstrating its potential to modulate interconnected biological processes and pathways. Its ability to target multiple proteins and mechanisms simultaneously supports its traditional use and suggests its potential for therapeutic applications in immune modulation, cancer therapy, metabolic disorders, and inflammatory conditions. Future experimental validation of these predictions will further elucidate their mechanisms and expand their potential clinical applications.

## CONCLUSION

The Tri-Ka-Tuk herbal compound demonstrates therapeutic potential *via* network pharmacology analysis by regulating critical biological pathways and molecular targets associated with inflammation, oxidative stress, metabolism, and apoptosis. Integrative network pharmacology clarifies its multi-target, multi-pathway processes, highlighting important functions in immune modulation, cancer progression, and metabolic diseases. These findings link traditional Thai medicine with current pharmacological knowledge, highlighting the importance of experimental validation to verify its efficacy and improve its therapeutic applications. Future research should focus on experimental validation through molecular docking simulations to confirm compound-target binding,* in vitro *cell-based assays to evaluate bioactivity, and *in vivo* animal studies to assess pharmacokinetics and therapeutic effects. Clinical trials should also be considered to translate these findings into practical applications. Additionally, multi-omics approaches, including metabolomics and transcriptomics, can be integrated to provide deeper insights into the mechanisms of Tri-Ka-Tuk’s therapeutic potential.

## Figures and Tables

**Table 1:** Phytochemicals identified in the herbal extracts of the Tri-Ka-Tuk formula utilized for network pharmacology investigation.

**Plant Species**	**Phytochemical Compound**	**Pharmacological Studies**	**References**
*Piper nigrum *Linn.	**1. Amides/ Alkaloids (Piperidine or Piperidine-derived)** Brachyamide, Guineensine, Pentadienoyl as Piperidine Isobutyleicosadienamide, Isobutyl-eicosatrienamide, Retrofractamide A, Retrofractamide B, Pellitorine, Piperanine, Pipercallosine, Dehydropipernonaline, Sarmentosine, Sarmentine, Piperonylamine, Chavicine, Piperine, Isochavicine, Isopiperine, Piperlonguminine **3. Lactams** Piperolactam D, Cepharadione A **4. Ketones** 10-Tricosanone **5. Phenylpropanoids** Feruperine, Piperettine **6. Terpenes** β-Caryophyllene, Limonene, Sabinene, α-Pinene, α-Humulene, α-Copaene, α-Cadinol, α-Thujene, β-Pinene, β-Phellandrene, Copaene, Myrcene, Linalool, Sylvestrene, Germacrene D, β-Myrcene, Isoterpinolene **7. Miscellaneous** Tricholein, Trichostachine, Paprazine	Antioxidant, antiobesity, antitumor, antipyretic, anticonvulsant, antithyroid, antifungal, antibacterial, insecticidal, hepatoprotective, antiasthmatic, larvicidal, antihypertensive, antiinflammatory, antidiabetic, antidiarrheal, bioavailability enhancer, immunomodulatory, antiepileptic, antifertility, GI stimulant, lipid metabolism accelerator, anticancer, CNS stimulant, diuretic, aphrodisiac, blood purifier and antiplatelet, anti-inflammatory activity, cosmoperine activity, antidepressants activities, antimicrobial, anti-obesity, neuroprotective properties, gastroprotective, and insecticidal activities	[61-66]
*Zingiber officinale* Roscoe.	**1. Aldehydes** Geranial, Neral, β-Citronellal, Hexanal, Decanal, Citronella, (Z)-3,7-Dimethylocta-3,6-dienal, (E)-3,7-Dimethylocta-3,6-dienal, (E)-Dodec-2-enal, Methyl heptanone **2. Ketones** 6-Gingerol, 8-Gingerol, 10-Gingerol, Zingerone, Gingerenone-A, 6-Dehydrogingerdione, 6-Gingerdione, 8-Gingerdione, 10-Gingerdione, 4-Shogaol, 6-Shogaol, 8-Shogaol, 10-Shogaol, 12-Shogaol, Methyl-8-shogaol 6-Paradol, 10-Paradol, 2-Heptanone, 2-Nonanone, 2-Undecanone, β-Cyclocitral **3. Alcohols** Citronellol, Geraniol, Nerol, Myrtenol, Borneol, endo-Borneol, 2-Decanol, 3,7-Dimethyloct-6-en-1-yn-3-ol 3,7-Dimethylocta-1,6-dien-3-ol, 2-Heptanol, 2-Nonanol cis-Sesquisabinene hydrate, trans-Sabinene hydrate, (1R)-(–)-Myrtenal **4. Phenols/Polyphenols** Quercetin, Isoeugenol, Dehydrozingerone, (E)-7-(3′,4′-Dihydroxyphenyl)-1-(4″-hydroxy-3″-methoxyphenyl)hept-4-en-3-one, 5-Hydroxy-7-(4′-hydroxy-3′,5′-dimethoxyphenyl)-1-(4″-hydroxy-3″-methoxyphenyl)heptan-3-one **5. Carboxylic Acids** Geranic acid, Tetracosanoic acid, 2,4-bis(3,4-Dihydroxyphenethyl)pentanedioic acid **6. Esters** L-Bornyl acetate, Neryl acetate, Geranyl acetate, Myrtenyl acetate, Ethyl acetate, Methyl acetate, Citronellyl acetate **7. Terpenes** Camphene, α-Terpinene, α-Terpineol, 4-Terpineol, β-Caryophyllene, Germacrene, β-Elemene, D-Limonene, α-Phellandrene, β-Phellandrene, Neoclovene, Thujopsene, β-Eudesmol, α-Eudesmol, Zingiberol, Zingiberenol allo-Aromadendrene, (–)-allo-Aromadendrene **8. Sulphides** Diethyl sulphide, Ethyl isopropyl sulphide, Methyl allyl sulphide **9. Miscellaneous** β-Sitosterol, Dibutyl phthalate, Methylbenzene, Styrene, o-Cymene, p-Cymene	Antioxidant, anti-inflammatory, antimicrobial, anticancer, neuroprotective, cardiovascular protective, respiratory protective, antiobesity, antidiabetic, antinausea, antiemetic activities, antitumor, hypoglycemic, lipid lowering, immune regulation, antivirus, antifatigue	[67-69]
*Piper retrofractum *Vahl.	**1. Amides/ Alkaloids** Pipernonaline, (2E,14Z)-N-Isobutyleicosa-2,14-dienamide, Piperine, Methyl piperate, Sylvatine, Piperlonguminine	Antifungal, antiobesity, antibacterial, antitubercular, antitubercular, antipyretic	[70-71]

## Data Availability

All the data and supporting information is provided within the article.

## LIST OF ABBREVIATIONS

PPI: Protein-Protein Interaction
ADME: Absorption, Distribution, Metabolism, and Excretion
P-gp: P-glycoprotein
MAPK: Mitogen-Activated Protein Kinase
MD: Molecular Dynamics
RCTs: Randomized Controlled Trials
MS: Mass Spectrometry
